# IL-37 counteracts inflammatory injury in the temporomandibular joint via the intracellular pathway

**DOI:** 10.3389/fphar.2023.1250216

**Published:** 2023-11-20

**Authors:** Jun Li, Sisi Peng, Ying Yan, Shan Yan, Xin Cao, Yong Li, Luying Zhu, Jie Xu

**Affiliations:** ^1^ College of Stomatology, Chongqing Medical University, Chongqing, China; ^2^ Chongqing Key Laboratory for Oral Diseases and Biomedical Sciences, Chongqing, China; ^3^ Chongqing Municipal Key Laboratory of Oral Biomedical Engineering of Higher Education, Chongqing, China; ^4^ Chongqing Emergency Medical Center, Chongqing, China; ^5^ Chongqing University Central Hospital, Chongqing, China

**Keywords:** intracellular IL-37, inflammation, temporomandibular joint, synovitis, osteoarthritis

## Abstract

**Background:** The temporomandibular joint is often afflicted by osteoarthritis (TMJOA), causing pain and dysfunction, which is particularly prevalent in the elderly population. IL-37 is effective in avoiding excessive inflammatory damage to the organism. This article investigates the role and mechanism of intracellular IL-37 in TMJOA.

**Methods:** Enzyme-linked immunosorbent assay, quantitative real-time polymerase chain reaction, Western blotting, Senescence-associated β-galactosidase staining, immunofluorescence, and lentivirus were performed to elucidate the underlying mechanism.

**Results:** The results confirmed that IL-37 in synovial cells decreased with aging. Inflammatory stimulus elevated intracellular IL-37 in synoviocytes, while lentiviral knockdown of IL-37 resulted in more inflammatory factor production. Dynamic changes of IL-37 were observed in the nucleus and supernatant. In addition, Caspease-1 inhibitor hindered intracellular IL-37 maturation, and Smad3 inhibitor caused the loss of nuclear translocation of mature IL-37. Transfection of synovial cells with IL-37-expressing lentivirus resulted in relief not only of synovitis but also of the cartilage damage and inflammation caused by synovitis.

**Conclusion:** This study provides new insights into the intracellular anti-inflammatory mechanism of IL-37. It also confirms that IL-37 decreases with cellular senescence and that increasing intracellular IL-37 can effectively treat synovitis and synovitis-induced inflammatory damage to cartilage.

## 1 Introduction

The temporomandibular joint (TMJ) is crucial in the maxillofacial region because it is involved in complex movements such as chewing, pronunciation and facial expression (Schellhas, 1989). However, TMJ is often affected by osteoarthritis, which causes pain and dysfunction in patients. TMJ Osteoarthritis (TMJOA) is a chronic painful disease whose pathological features mainly include degenerative cartilage changes, subchondral bone remodeling and synovitis (Cardoneanu et al., 2022; Wang et al., 2023). Although the etiology of TMJOA is multi-factorial and complex, continuous inflammatory stimulation is still one of the main reasons for TMJOA (Zhang et al., 2023; Zhong et al., 2023). TMJOA is age-related, and much research has confirmed that its incidence and severity increase with the increase of age (Bernhardt et al., 2007; Schmitter et al., 2010; Alzahrani et al., 2020). However, further studies on the high incidence of TMJOA in the aging population are still lacking.

The synovial membrane can produce and secrete a variety of nutrients to maintain the viscosity of the synovial fluid, nourish cartilage and protect the bone (Ulmner et al., 2022). However, activated synoviocytes in inflammatory synovitis produce and release a large number of pro-inflammatory factors when synovitis occurs, such as IL-1β, IL-6, MMPs and TNF-α, which mediate cartilage destruction and TMJ inflammation through various ways (Yuan et al., 2004; Sellam and Berenbaum, 2010; Jiang et al., 2016; Ulmner et al., 2022). Although synovitis is not a prerequisite for the occurrence of TMJOA, more and more studies have indicated that the pro-inflammatory mediators released by synovitis are critical for the occurrence and development of osteoarthritis and the destruction of articular cartilage. On the other hand, disrupted and decomposed cartilage generally leads to excessive production of proteolytic enzymes, which in turn aggravates the inflammation of synovial tissue, forming a positive feedback loop (Sellam and Berenbaum, 2010; Felson et al., 2016; Mathiessen and Conaghan, 2017; Liao et al., 2020).

As a natural inhibitor of immune response and inflammation, IL-37 is upregulated to avoid further development of inflammation when the body is stimulated. It has been demonstrated in various tissues, such as the myocardial, periodontal tissue, and respiratory tract (Feng et al., 2022; Sun et al., 2022; Li et al., 2023). In these inflammatory tissues, IL-37 effectively reduces the expression of inflammatory factors such as IL-1β, IL-6, TNF-α and IL-18, and the inflammatory damage of tissues. IL-37 is a dual function cytokine with both intracellular and extracellular anti-inflammatory properties. Extracellular IL-37 binds to IL-18Rα on the cell surface of chondrocytes and macrophages to exert its anti-inflammatory effect in TMJOA (Luo et al., 2019; Luo et al., 2020). However, the role and mechanism of intracellular IL-37 in the arthritis of TMJ are unclear.

In previous studies, we observed that the expression of IL-37 was most abundant in the synovial tissue of TMJ and concentrated in the nucleus through immunohistochemical staining (Luo et al., 2019). These findings suggest that the intracellular pathway of IL-37 may play an essential anti-inflammatory role in TMJ inflammation. Our study found that elderly TMJOA patients expressed less IL-37, which is present in synoviocytes as an inflammatory antagonist, compared to young adults. We also confirmed the therapeutic effect of intracellular IL-37 on TMJ inflammation and explored its anti-inflammatory pathway and mechanism. This study may provide a therapeutic target for the prevention and treatment of the inflammation associated with TMJ.

## 2 Materials and methods

### 2.1 Sample collection and synovial cell culture

1 mL of synovial fluid diluted with normal saline was extracted during intra-articular injections in 58 TMJOA patients (17–80 years old, mean age 44 years old). The specific articular synovial fluid extraction method was described previously. The synovial fluid was centrifuged at 2000 r/min for 10 min and then stored at −80°C.

Synovial tissue was obtained from patients undergoing TMJ condylectomy and arthroplasty. Synovialis collected after surgery was washed with PBS and immersed in DMEM/F-12 medium (Gibco, Carlsbad, CA, United States). The synovial was cut to approximately 1 mm in size using ophthalmic scissors. The scissored synovial blocks were evenly distributed at the bottom of the cell culture bottle, and then DMEM/F-12 complete medium containing 10% fetal bovine serum (Gibco) and 1% penicillin/*streptococcus* was added. The extracted synovial cells were cultured in a 37°C incubator containing 5% CO_2_. Chondrocytes were extracted and cultured the same way as before.

The above experiments were performed with patient consent and approved by the Ethics Committee of Chongqing Medical University. Samples were collected in compliance with the Declaration of Helsinki.

### 2.2 Enzyme-linked immunosorbent assay (ELISA)

Cells were treated accordingly and the supernatant was retained and then centrifuged at 4,000 rpm for 20 min The supernatant obtained was used for experiments immediately or stored at −80°C. The supernatant was used to measure the expression of IL-37 in the cells. The ELISA kit for measuring IL-37, IL-6, MMP9, and MMP13 in cell supernatants was purchased from Jianglai Biotech (Shanghai, China). All experiments were performed according to the manufacturer’s instructions. The website is http://www.laibio.com/. The absorbance of the supernatants was evaluated at 450 nm in an enzyme labeling instrument (Molecular Devices, Shanghai, China).

### 2.3 Senescence-associated β-galactosidase (SA-β-gal) staining

Synovial cells were inoculated in 6-well plates when grown to the first or seventh generation, respectively. After 12 h, the plates were washed once with PBS, and 1 mL of β-galactosidase staining fixative (Beyotime, Shanghai, China) was added to each well to fix the cells for 15 min 1mL of staining working solution was added to each well and then incubated at 37°C for 12 h. Finally, the staining results were observed and recorded under a microscope.

### 2.4 Quantitative real-time polymerase chain reaction (qRT-PCR)

For gene expression analysis, total RNA was extracted from synovial cells using RNAiso plus (Takara, Nogihigashi, Japan). RNA was reverse transcribed using PrimeScript™ RT Reagent Kit with gDNA Eraser (Takara) to obtain cDNA. real-time PCR was carried out using Power TB Green PCR Master Mix (Takara). The expression of data was evaluated by the 2-^△△Ct^ method. Primer sequences used in the article are displayed in Table 1.

**TABLE 1:** Primer sequences for RT-q PCR.

Gene	Forward primer (5′-3′)	Reverse primer (5′-3′)
GAPDH	TGC​ACC​ACC​AAC​TGC​TTA​G	GATGCAGGGATGATGTTC
IL-37	TTG​CAT​TAG​CCT​CAT​CCT​TGA	GGC​GTG​CTG​ATT​CCT​TTT​G
IL-6	AGCCCACCGGGAACGA	GGACCGAAGGCGCTTGT
TNF-α	TCA​TCT​ACT​CCC​AGG​TCC​TCT​TCA	TCT​GGC​AGG​GGC​TCT​TGA​TG
MMP 9	CCC​TTG​TGC​TCT​TCC​CTG​GA	TCT​GCC​ACC​CGA​GTG​TAA​CC
MMP13	ATT​AAG​GAG​CAT​GGC​GAC​TTC​T	CCC​AGG​AGG​AAA​AGC​ATG​AG

### 2.5 Western blotting (WB)

Total intracellular proteins were extracted using RIPA, and nuclear or cytoplasmic proteins were obtained using a Nuclear and Cytoplasmic Protein Extraction Kit (Beyotime). For Western blot analysis, 30 μg of cell lysates were loaded for each sample. Then, the proteins were separated in SDS-PAGE gels and transferred to PVDF membranes (Millipore). After blocking with 5% skimmed milk in TBST, the proteins were blotted with antibodies for IL-37 (1:1,000; Abcam, ab153889) and Proteintech, 11863-1-AP), p-Smad3 (1:1,000; CST, 9520S), IL-6 (1:1,000; CST, 12153S), TNF-α (1:1,000; Proteintech, 17590-1-AP), MMP9 (1:1,000; Bioss, bs-22502R), MMP13 (1:1,000; Abcam, ab39012), GAPDH (1:1,000; HUABIO, ET1601-4), Histone-H3 (1:1,000; Proteintech, 17168-1-AP) at 4°C overnight, followed by incubating with HRP- labeled secondary antibodies (1:5,000; HUABIO, HA1001) for 2 h. Images were obtained and analyzed by a computer program (Bio-Rad, United States).

### 2.6 Transfection of lentivirus

Synovial cells were transfected with lentiviruses (Tsingke Biotech, Beijing, China) expressing or targeting IL-37 with the help of Polybrene (Beyotime) according to the manufacturer’s instructions (https://www.tsingke.com.cn/). The multiplicity of infection (MOI) of synoviocytes is 20; Virus concentration of 2×10^6^ transducing units/mL. At 48 h after lentivirus transfection, synoviocytes were treated with Puromycin Dihydrochloride (1 μg/mL, Beyotime) for 1 week to remove transfection failures. Subsequent treatments were started after 48 h of ordinary culture.

### 2.7 Immunofluorescence

Synovial cells were treated with IL-1β (10 ng/mL) for 12 h to induce intracellular expression of p-Smad3 and IL-37. After washing the cells with PBS, the cells were fixed with 4% paraformaldehyde and closed with goat serum for 1 h. Next, cells were incubated overnight with p-Smad3 primary antibody (1:200; CST, 9520S) and the following day with Alexa Fluor 594-coupled goat anti-rabbit antibody (1:500; Abcam, ab150080) for 1 h. Cell nuclei were stained with DAPI. Images were observed under a fluorescent microscope.

### 2.8 Statistical analysis

The data were statistically analyzed by GraphPad Prism 8 (GraphPad Software Inc., La Jolla, CA). Statistical differences were analyzed by *t*-test or one-way ANOVA. For condylar chondrocytes, experiments were conducted in triplicatewith cells from three donors, and data were statistically significant if *p* < 0.05.

## 3 Results

### 3.1 Reduced IL-37 expression in senescent sy novial cells

**FIGURE 1:**
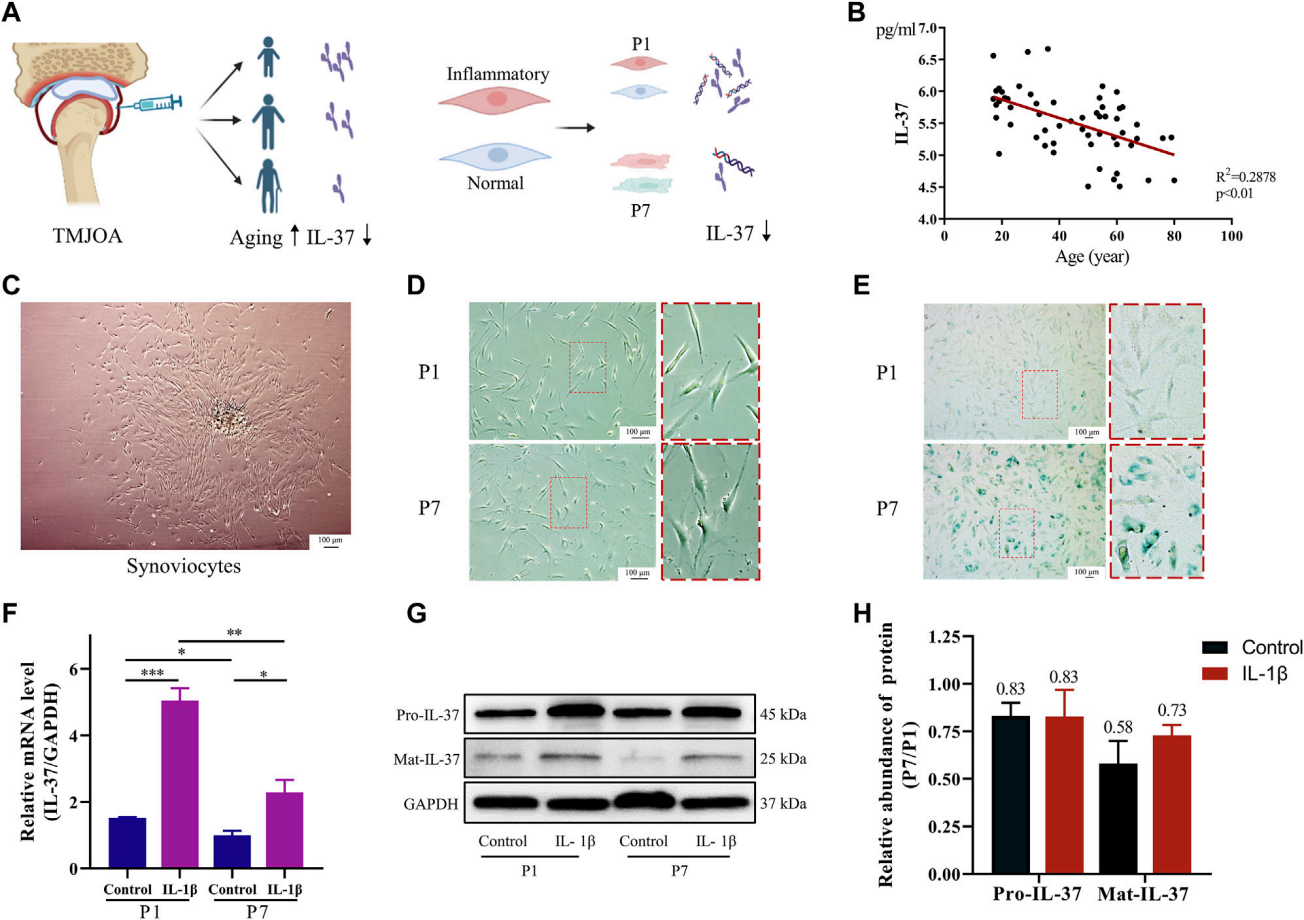
Reduced IL-37 expression in senescent sy novial cells. **(A)** Diagram of synovial fluid and intracellular IL-37 detection. **(B)** IL-37 in synovial fluid of 58 TMJOA patients was detected by ELISA. **(C)** Primary synovial cells were extracted by explant cultures. **(D)** Observation of the morphology of P1 and P7 under electron microscope. **(E)** SA-β-gal staining of P1 and P7 synovial cells, and aging synovial cells appear dark blue. Synovial cells were stimulated with IL-1β (10 ng/mL) for 24 h. PCR **(F)** and Western blot **(G)** were performed to detect intracellular IL-37. **(H)** Quantitative analysis of Western blot. The numbers above the bars indicate fold changes. Results are expressed as the mean ± S.E.M (n = 3). *p < 0.05; **p < 0.01; ***p < 0.001.

At first, IL-37 was analyzed by ELISA in the synovial fluid of 58 TMJOA patients. The results revealed that IL-37 decreased with age in TMJOA patients, indicating that older patients have a reduced ability to produce IL-37 (Figure 1B). Further studies extracted primary synovial cells by tissue-sticking method. The results showed short spindle-shaped synovial cells crawling out from within the synovial tissue (Figure 1C). Then, the synovial cells were passaged to the first and seventh generations (P1 and P7) to simulate synovial tissue in the young and aging states. Microscopically, it was observed that P7 became larger and showed more bifurcation compared to P1 (Figure 1D). Also, β-galactosidase staining confirmed that P7 had darker blue staining and a greater proportion of stained cells than P1 (Figure 1E). When P1 and P7 were stimulated with IL-1β to simulate the inflammatory state of young and old synovium, the expression of IL-37 precursor (Pro-IL-37) and mature IL-37 (Mat-IL-37) in P7 was significantly reduced both at rest and when stimulated with IL-1β (Figures 1F–H).

### 3.2 IL-37 exerts anti-inflammatory effects

**FIGURE 2:**
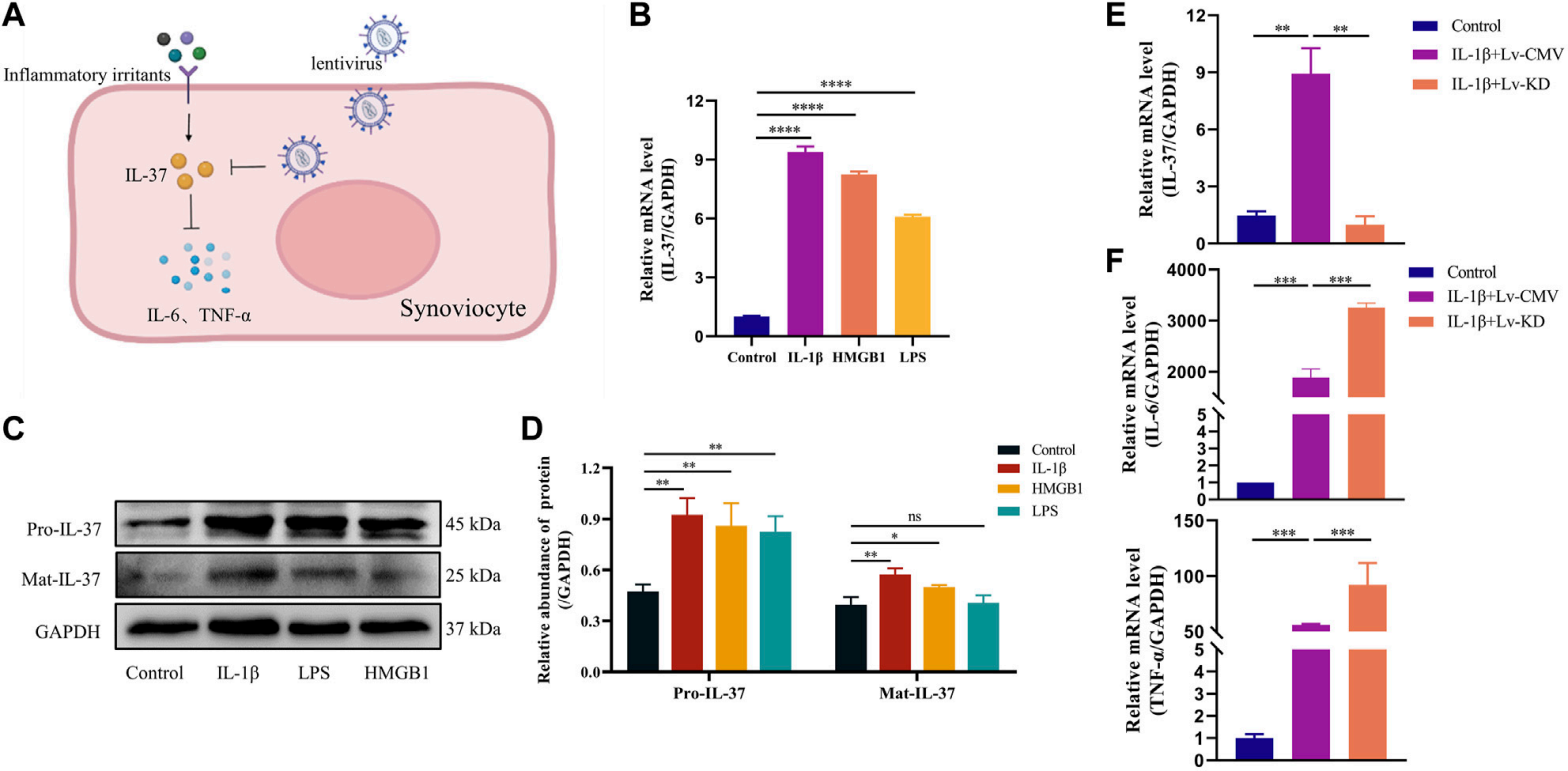
IL-37 exerts anti-inflammatory effects. **(A)** Diagram of il-37 against inflammation. IL-1β (10 ng/mL), HMGB1 (10 ng/mL) and LPS (1 μg/mL) stimulated synovial cells for 24 h. The levels of protein **(B)** and RNA **(C)** were detected. **(D)** Quantitative analysis of Western blot. IL-1β was stimulated for 24 h after transfection of synovial cells with IL-37 knockdown lentivirus (Lv-KD) and control lentivirus (Lv-CMV). PCR was used to detect the RNA levels of IL-37 **(E)**, IL-6 and TNF-α **(F)** in Synovial cells. Results are expressed as the mean ± S.E.M (n = 3). *p < 0.05; **p < 0.01; ***p < 0.001; ****p < 0.0001; ns, not significant.

Synovial cells were stimulated with inflammatory factors (IL-1β, HMGB1, LPS) to mimic the state of synovial tissue during inflammatory stimulation, which resulted in a significant increase of IL-37 in synovial cells at the protein and gene levels (Figures 2B–D). To verify the anti-inflammatory effect of IL-37 in synoviocytes, we knocked down IL-37 with lentivirus, which resulted in a Remarkable reduction of IL-37 (Figure 2E). In response to IL-1β stimulation, IL-6 and TNF-α were elevated in synovial cells. However, silencing of intracellular IL-37 using lentivirus resulted in an additional elevation of IL-6 and TNF-α (Figure 2F).

### 3.3 Dynamic changes of IL-37 inside and outside the cell

**FIGURE 3:**
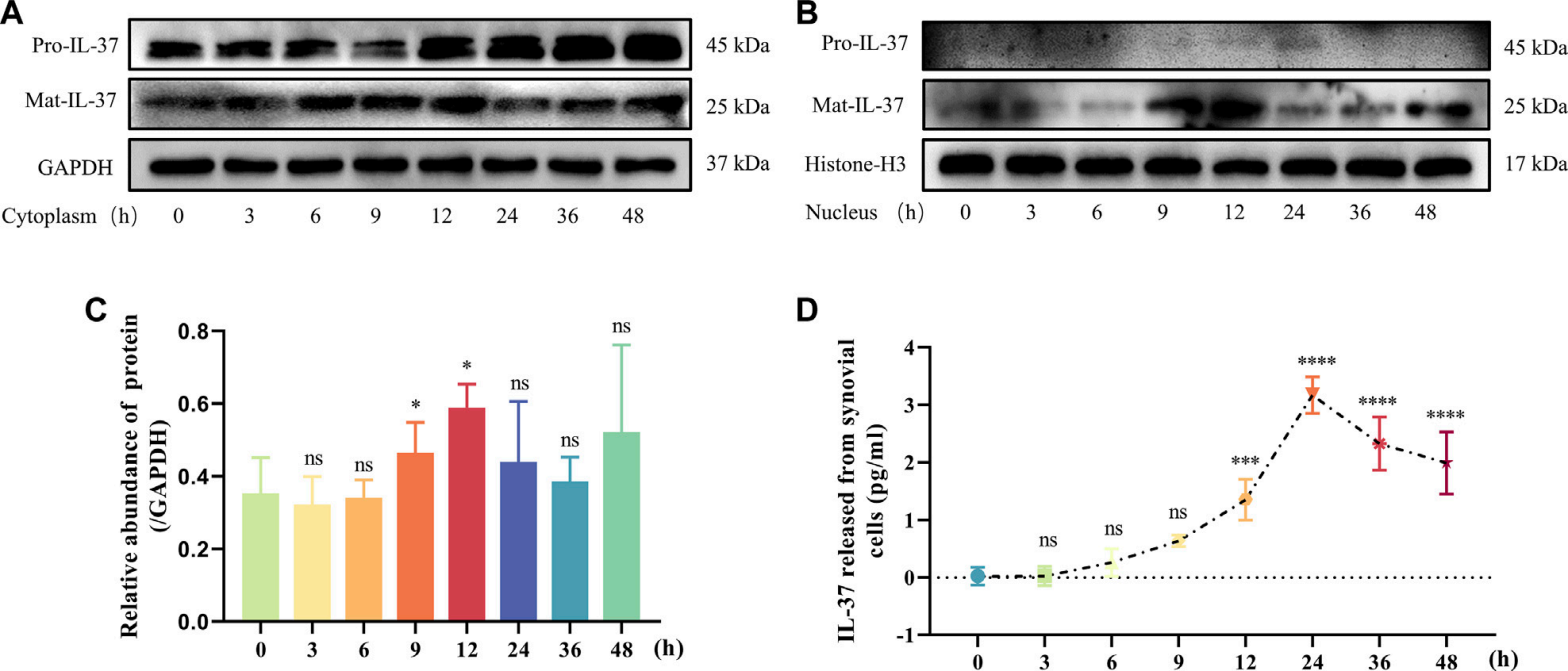
Dynamic changes of IL-37 inside and outside the cell. Samples were collected at 3, 6, 9, 12, 24, 36, and 48 h after treatment with IL-1β. IL-37 in cytoplasm **(A)** and nucleus **(B)** were detected by Western blot. **(C)** Quantitative analysis of Mat-IL-37 in the nucleus. **(D)** Cell supernatants were collected after treatments. ELISA was used to detect the content of IL-37. Results are expressed as the mean ± S.E.M (n = 3). Ns, not significant; ***p < 0.001; ****p < 0.0001 for treatment group compared to control group.

IL-37 is a bifunctional anti-inflammatory cytokine, so we examined the dynamic changes of IL-37 inside and outside the cells after 0–48 h stimulation of synovial cells using IL-1β. Within the cytoplasm, Pro-IL-37 exhibited a consistently elevated trend. Although Mat-IL-37 also showed an elevated trend in the cytoplasm, the most significant elevation occurred at 6–12 h and started to decrease after 12 h (Figure 3A). In the nucleus, No visible expression of Pro-IL-37 can be observed. On the other hand, Mat-IL-37 showed a tendency of elevation followed by a decrease, and the most pronounced upward shift occurs at 9 and 12 h (Figures 3B, C). ELISA detected a slow release of extracellular IL-37 into the extracellular compartment starting at 6 h, reaching a maximum at 24 h, and following a decline (Figure 3D).

### 3.4 Mechanism of intracellular IL-37 maturation and nuclear translocation in synoviocytes

**FIGURE 4:**
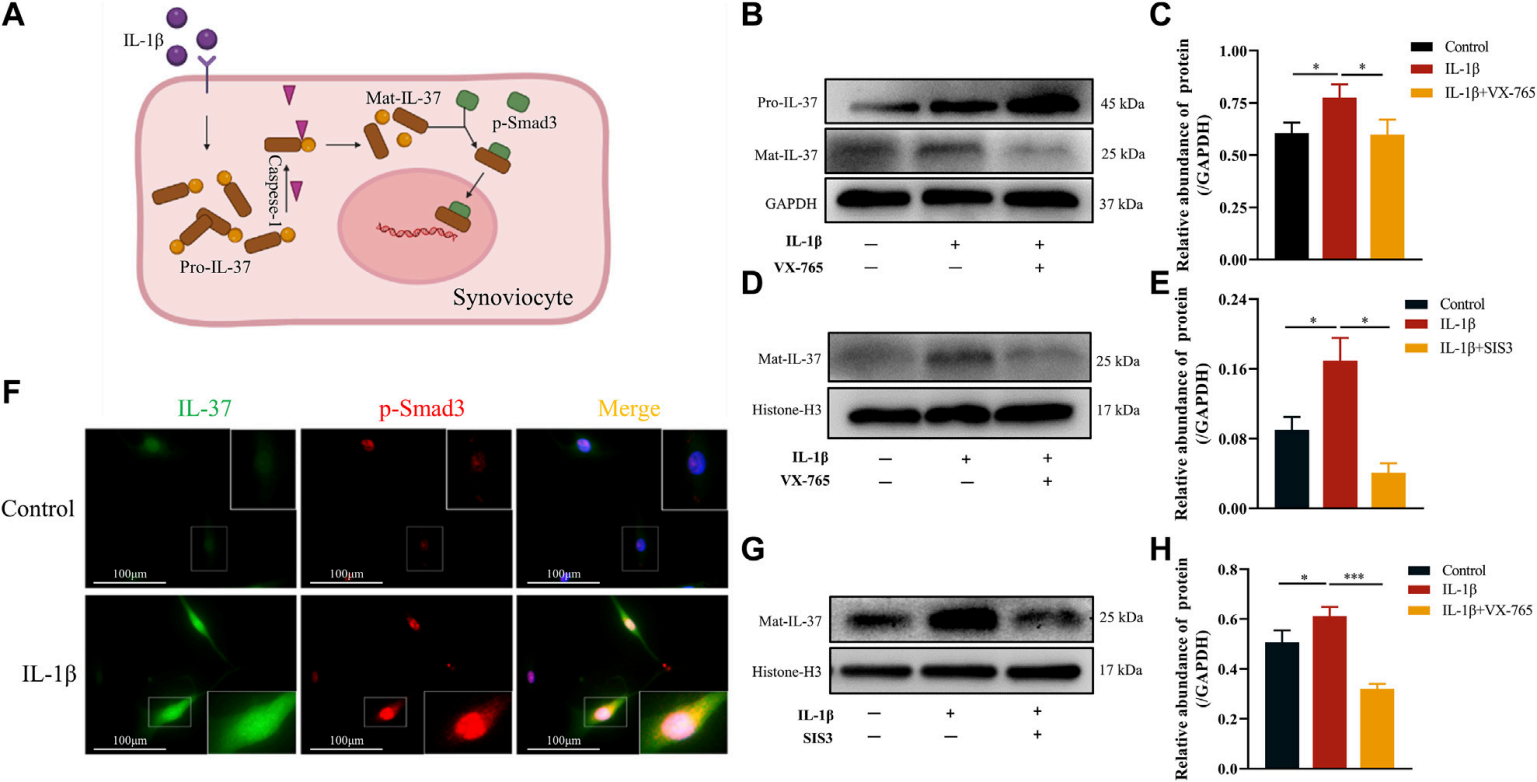
Mechanism of intracellular IL-37 maturation and nuclear translocation in synoviocytes. **(A)** Schematic diagram of Mat IL-37 translocation into the nucleus. Synovial cells were pretreated with Caspase-1 (VX-765, 10 μM) and p-Smad3 (SIS3, 10 μM) inhibitors for 2 h prior to IL-1β treatment for 12 h. Then Western blot was used to detect IL-37 protein levels in whole cell lysates **(B,C)** and nuclear lysates **(D,E) (G,H)**. **(F)** The synovial cells were transfected with lentivirus to express IL-37 with green fluorescent label. Then, the intracellular distribution of IL-37 and p-Smad3 (red) was observed by immunofluorescence after 12 h treatment with IL-1β. The nuclei are stained by DAPI. Results are expressed as the mean ± S.E.M (n = 3). *p < 0.05; ***p < 0.001.

Upon exposure to stimuli, IL-37 is produced intracellularly as Pro-IL-37. The Pro-IL-37 becomes Mat-IL-37 after cleavage by Caspase-1, as pretreatment with Caspase-1 inhibitor (VX-765) almost completely inhibited the IL-1β-triggered elevation of Mat-IL-37. In contrast, the Pro-IL-37 exhibited elevated (Figures 4B, C). On the other hand, analysis of IL-37 levels in the nucleus revealed that VX-765 curtailed the increased nuclear translocation of Mat-IL-37 caused by IL-1β stimulation (Figures 4D, E).

Regarding the mechanism of nuclear translocation of IL-37, synoviocytes transfected with lentivirus expressing IL-37 labeled by green fluorescent. Upon stimulation with IL-1β, intracellular expression of both IL-37 and p-Smad3 upregulated. At the same time, the intracellularly abundantly expressed IL-37 co-localized with p-Smad3 in the nucleus and perinucleus, especially in the nucleus (Figure 4F). In contrast, pretreatment of synoviocytes with SIS3 attenuated or even eliminated IL-37 nuclear translocation induced by IL-1β stimulation (Figures 4G, H).

### 3.5 Intracellular IL-37 inhibits synovial inflammation

**FIGURE 5:**
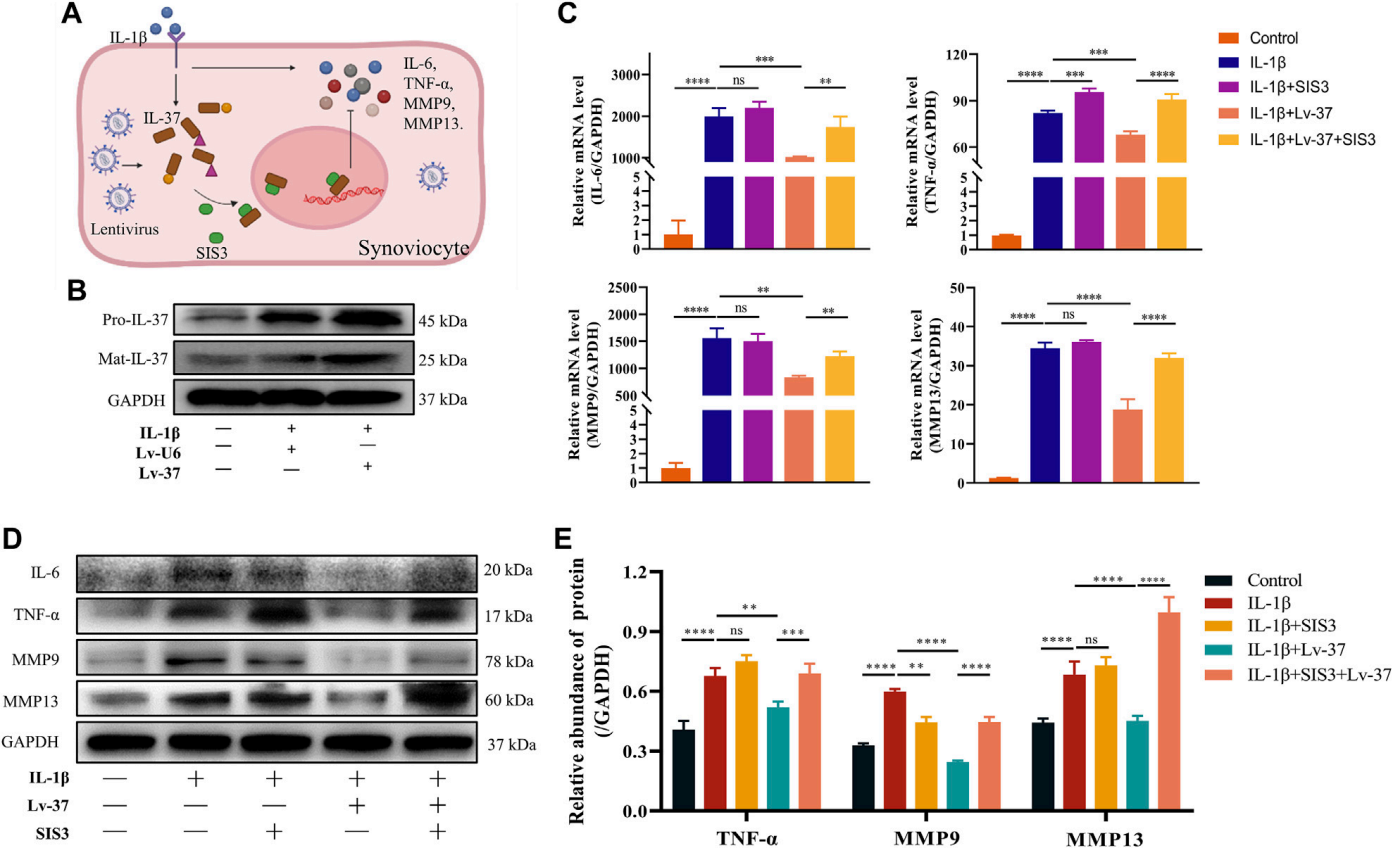
Intracellular IL-37 inhibits synovial inflammation. **(A)** Schematic illustration of Anti-inflammatory mechanism of intracellular IL-37. IL-37 was overexpressed in synovial cells with NC (Lv-U6) or IL-37 (Lv-37) lentivirus. Then SIS3 was pretreated for 2 h before IL-1β (12 h) treatment. **(B)** Western blot observed intracellular IL-37 levels. The protein and RNA levels of IL-6, TNF-α, MMP9, and MMP13 were analysed by PCR **(C)** and Western blot **(D)**. **(E)** Quantitative analysis of Western blot. Results are expressed as the mean ± S.E.M (n = 3). *p < 0.05; **p < 0.01; ***p < 0.001; ****p < 0.0001; ns, not significant.

To verify if intracellular IL-37 can exert anti-inflammatory effects, lentivirus transfected synovial cells to obtain intracellular overexpression of IL-37. Increased production of IL-37 in lentivirus-transfected synoviocytes at the protein level was observed (Figure 5B). Although IL-1β stimulation caused an increase of IL-6, TNF-α, MMP9, and MMP13, the production of the above-mentioned inflammatory factors received significant inhibition when intracellular IL-37 was abundantly expressed (Figures 5C–E). Smad3 is a protein necessary for IL-37 nuclear translocation to exert anti-inflammatory effects. When Sma d3 was inhibited by SIS3, intracellular IL-37 resistance to inflammatory factors is lost.

### 3.6 Synoviocyte conditioned medium regulates the inflammatory response in chondrocytes

**FIGURE 6:**
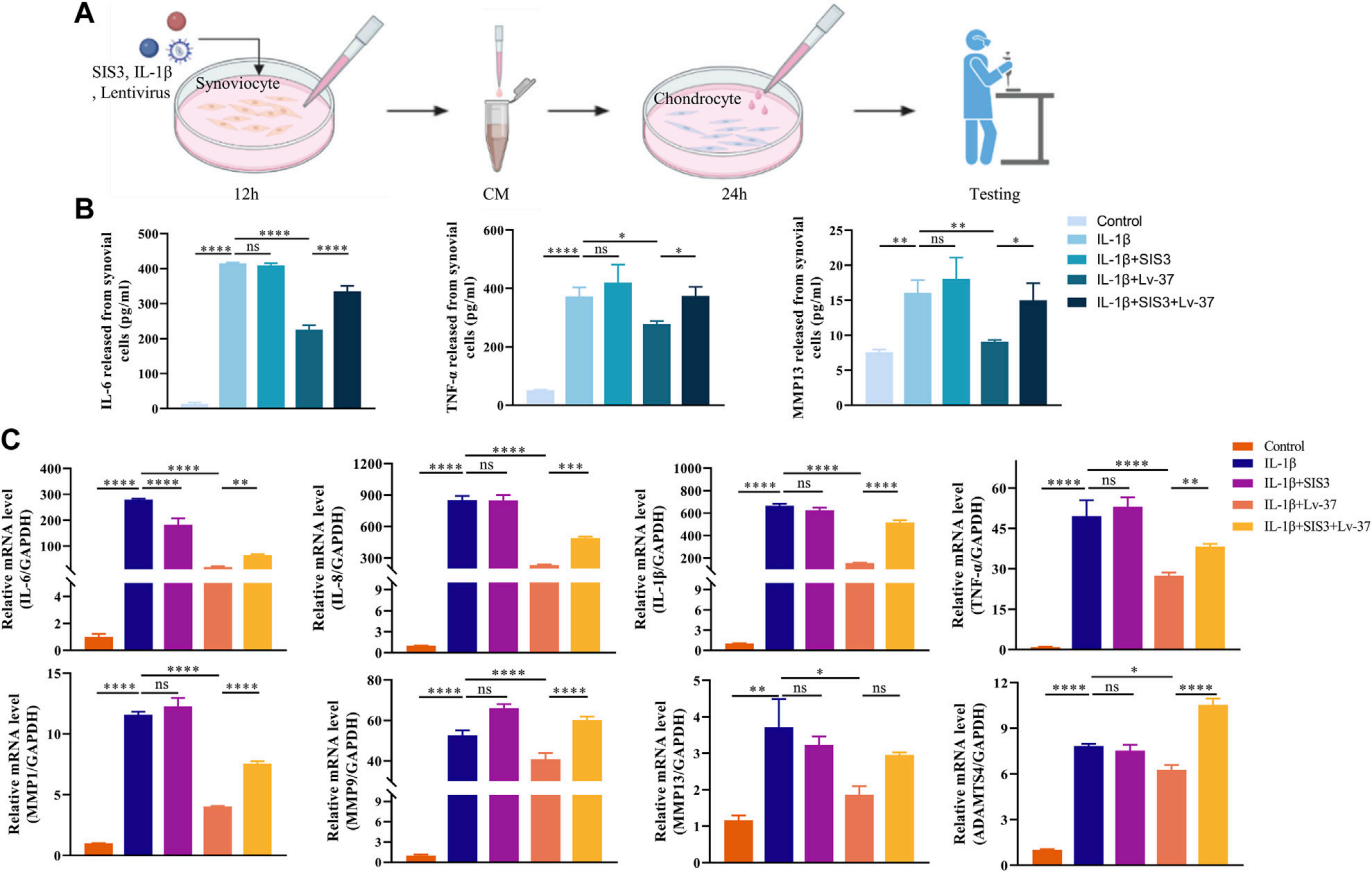
Synoviocyte conditioned medium regulates the inflammatory response in chondrocytes. **(A)** Conditioned media (CM) of synovial cells were collected for cartilage culture. The synovial cells were transfected with lentivirus expressing IL-37, After pretreated with SIS3 for 2h, synoviocytes stimulated by IL-1β for 12 h. Chondrocytes were cultured in the CM form synovial cells for 24 h. **(B)** The levels of sIL-6, TNF-α, MMP9, and MMP13 in supernatant were sdetected by ELISA. **(C)** PCR was conducted to measure IL-6, IL-8, IL-1β, TNF-α, MMP1, MMP9, MMP13, and ADAMTS4 in chondrocytes. Results are expressed as the mean ± S.E.M (n = 3). *p < 0.05; **p < 0.01; ***p < 0.001; ****p < 0.0001; ns, not significant.

During the development of TMJOA, inflamed synovium tends to release multiple inflammatory factors leading to the progression of osteoarthritis. We, therefore, collected conditioned medium (CM) from synovial cells (Figure 6B). ELISA was performed to detect the water of inflammatory factors in the supernatant. The results showed that in response to IL-1β stimulation, synovial cells released more IL-6, TNF-α and MMP13 (Figure 6C). At the same time, intracellular IL-37 significantly inhibited the release of these inflammatory factors. When chondrocytes were cultured using CM with synovial cells, we found that the supernatant of inflamed synovial cells also triggered chondrocyte inflammation, resulting in higher levels of IL-6, IL-8, IL-1β, TNF-α, MMP1, MMP9, MMP13, and ADAMTS4. However, when the intracellular IL-37 in synovial cells was increased, CM from inflammatory synoviocytes showed remarkably reduced inflammation induction in chondrocytes.

## 4 Discussion

The incidence and severity of TMJOA increase significantly with aging, which is confirmed in a large body of literature (Bernhardt et al., 2007; Schmitter et al., 2010; Alzahrani et al., 2020). Although the etiology of TMJOA is complex and multifactorial, inflammatory stimuli still play an essential role in its development, such as IL-6, TNF-α, IL-1β, and MMP13 (Yuan et al., 2004; Sellam and Berenbaum, 2010; Jiang et al., 2016; Ulmner et al., 2022). Given the strong anti-inflammatory effect of IL-37, we speculated whether the high prevalence of TMJOA in the elderly population was related to the reduced expression of IL-37. We, therefore, examined the level of IL-37 in the synovial fluid of patients with TMJOA and found that it decreased with aging. This initially verified our speculation.

The components of synovial fluid are mainly derived from synovial tissue, which includes various inflammatory irritants that contribute to cartilage destruction (Wang et al., 2015; Qi et al., 2020). In recent years, attention has begun to be paid to the importance of synovium in the etiology of OA, and some studies have even proposed synovitis as an independent risk factor for OA (Felson et al., 2016; MacFarlane et al., 2019; Wang et al., 2022). And we found much higher levels of IL-37 in the synovial membrane than in the articular disc, cartilage and condyle (Luo et al., 2019). This evidence led us to select synovium for further study. First- and seventh-generation synovial cells were then cultured to mimic the inflammation of young and old synovium. The results confirmed that P7 exhibited significant senescence and a reduced ability to produce IL-37 compared to P1. This further confirms our speculation that low intracellular of IL-37 may be responsible for the development of synovitis in senescent synoviocytes, which is detrimental to both the development and progression of TMJOA.

IL-37 counteracts inflammatory progression in the heart, periodontal and respiratory tracts (Feng et al., 2022; Sun et al., 2022; Li et al., 2023). In a synovitis cell model constructed using IL-1β, HMGB1, and LPS, intracellular IL-37 was responsively upregulated (more pronounced with stimulation of IL-1β). However, more pronounced synovitis was observed when IL-37 was knocked down using lentivirus. The above experiments confirm that IL-37 is generated as an inflammatory stimulus counteractant when the synovium is subjected to inflammatory stimuli. However, the knockdown of IL-37 using lentivirus decreases both intracellular and extracellular IL-37, which results in the inhibition of the anti-inflammatory effects of both intracellular and extracellular il-37. Our previous study found that IL-37 in inflamed joints is predominantly concentrated intracellularly, suggesting an important role for the intracellular anti-inflammatory pathway of IL-37 (Luo et al., 2019). Therefore, whether there is an anti-inflammatory effect of intracellular IL-37 still needs further confirmation.

The intracellular anti-inflammatory effect of IL-37 requires entry into the nucleus (Sharma et al., 2008; Bulau et al., 2014). When looking at IL-37 in and out of cells under inflammatory stimuli, we found that IL-37 was the first to translocate to the nucleus and then released into the extracellular space. This suggests that intracellular IL-37s are the first to exert anti-inflammatory effects in response to inflammatory stimuli. Furthermore, the intracellular anti-inflammatory mechanism of IL-37 appears to be achieved through its mature form, as no significant pro-IL-37 was observed in the nucleus.

Further, we explored the mechanism of IL-37 maturation and nuclear translocation in synovial cells. Consistent with previous studies (Kumar et al., 2002; Bulau et al., 2014), Casepase-1 was involved in the maturation process of IL-37, as its inhibitor significantly decreased the expression of Mat-IL-37. Furthermore, we observed that VX-765 impeded the movement of Mat-IL-37 toward the nucleus. We speculate that this may be because VX-765 reduces the total intracellular level of Mat-IL-37. The presence of Smad3 is necessary for the intracellular anti-inflammatory process of IL-37 (Nold et al., 2010; Luo et al., 2017), but the exact mechanism is still unknown. Our study also observed fluorescent co-localization of p-Smad3 with IL-37 in the nucleus and perinucleus. Importantly, we confirmed that SIS3 reversed the IL-1β-induced nuclear translocation of Mat-IL-37. This suggests that the role of p-Smad3 is to assist in the nuclear translocation of IL-37 during its intracellular anti-inflammatory process.

After elaborating on the mechanism of intracellular IL-37 maturation and nuclear translocation, we explore whether intracellular IL-37 is effective against synovitis. Synovial cells transfected with IL-37-expressing lentivirus significantly inhibited IL-6, TNFα, MMP9, and MMP13 upregulation caused by IL-1β. Notably, this resulted in both increased IL-37 secretion as well as increased extracellular anti-inflammatory effects. Therefore, we blocked the intracellular anti-inflammatory pathway of IL-37 using SIS3, which reversed the anti-inflammatory effect of intracellular IL-37. It confirms that intracellular IL-37 can effectively counteract inflammation and alleviate tissue damage caused by excessive inflammation.

Numerous studies have shown that synovitis occurs well before the onset of OA and releases various inflammatory factors promoting OA procession (Loeuille et al., 2005; Scanzello and Goldring, 2012; Hügle and Geurts, 2017; Liao et al., 2020). To further confirm the role of synovitis in promoting osteoarthrosis, we collected conditioned media from inflamed synovial cells. ELISA showed that inflamed synovium released more IL-6, TNF-α, and MMP13. These are all undesirable stimuli leading to cartilage breakage and the progression of osteoarthritis (Wang and He, 2018; Wang et al., 2020). Meanwhile, factors such as IL-6, IL-8, IL-1β, TNF-α, MMP1, MMP9, MMP13, and ADAMTS4 were elevated when chondrocytes were stimulated by CM from inflammatory synovium. These factors play a role in promoting cartilage destruction and osteoarthritis progression (Burrage et al., 2006; Latourte et al., 2017; Wang and He, 2018; Fei et al., 2019; Li et al., 2022). In contrast, when IL-37 levels are elevated in synovial cells, inflamed synovium will release fewer inflammatory factors and have significantly less damage to chondrocytes and induction of inflammation.

## 5 Conclusion

**FIGURE 7:**
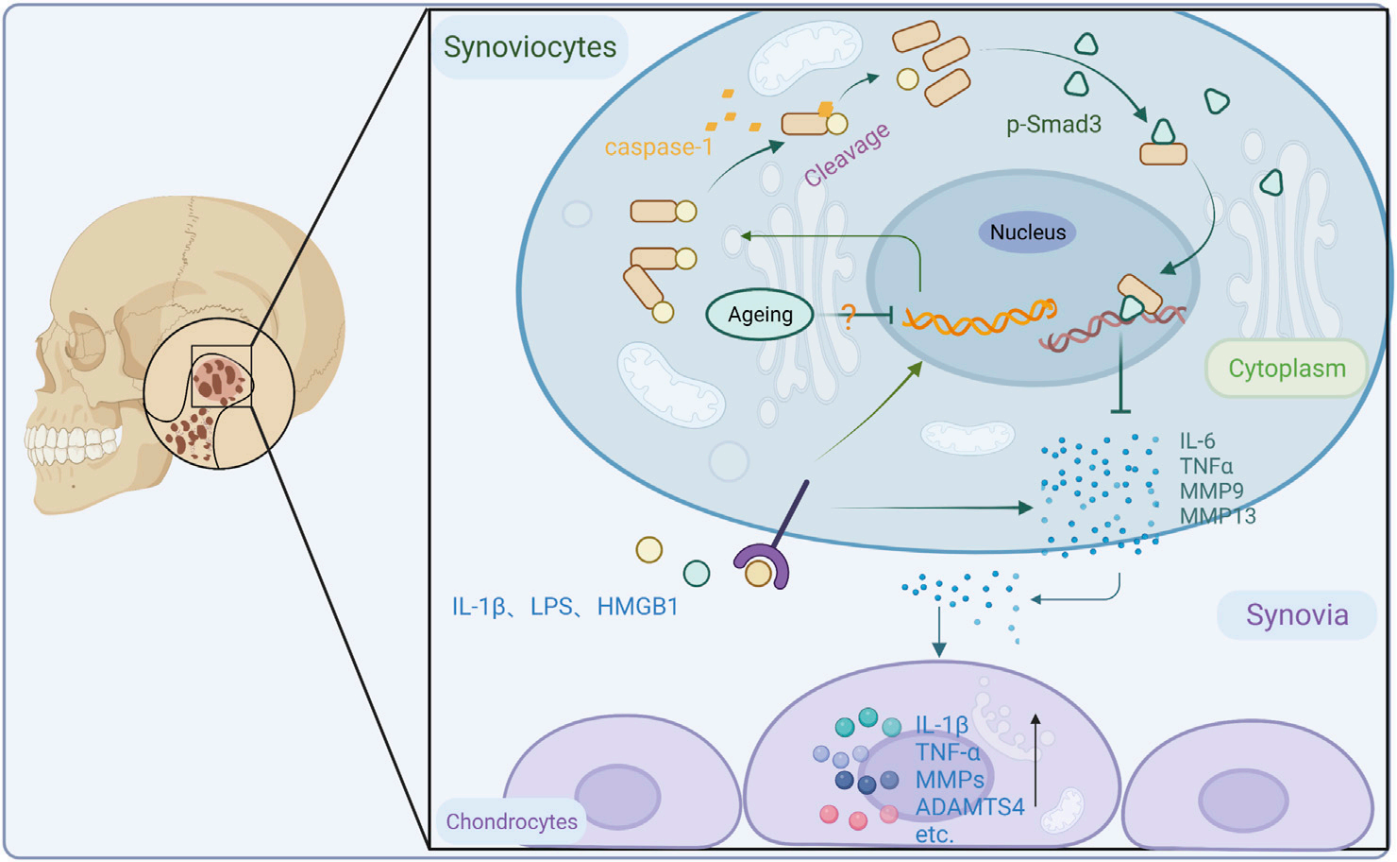
An illustration of intracellular IL-37 against arthritis in TMJ. In response to the stimulation of inflammatory factors, IL-37 is upregulated responsively in synovial cells in the form of precursors. Pro-IL-37 matures after cleavage by caspase-1 and translocates to the nucleus mediated by Smad3. This avoids excessive inflammatory responses in synovial cells and attenuates the release of IL-6, TNF-α, MMP9, and MMP13 and the resulting osteoarthritis.

In summary, our study confirm that the high prevalence of TMJOA in the elderly population is associated with reduced IL-37 levels. Restoring intracellular IL-37 levels effectively reduces synovial tissue inflammation and alleviates the destruction of cartilage and progression of inflammation caused by synovitis (Figure 7). This study provides new ideas on the anti-inflammatory mechanism of intracellular IL-37 and new insights into the etiology and treatment of TMJOA.

## Data Availability

The original contributions presented in the study are included in the article/Supplementary Material, further inquiries can be directed to the corresponding authors.
