# Supplementary material for: IL-37 counteracts inflammatory injury in the temporomandibular joint via the intracellular pathway
Source: Front Pharmacol. 2023 Nov 20;14:1250216. doi: 10.3389/fphar.2023.1250216 (PMC10694265; doi:10.3389/fphar.2023.1250216)
Supplement: Supplementary file 1 [file DataSheet1.ZIP › original blots - .pdf]

Since the molecular weights of Pro-IL-37, GAPDH, and Mat-IL-37 are 45kDa, 37kDa, and 25kDa, the PVDF membrane was cut into 3 parts separately for antibody incubation.

The names of the lanes are in left-to-right order

Figure. 1G

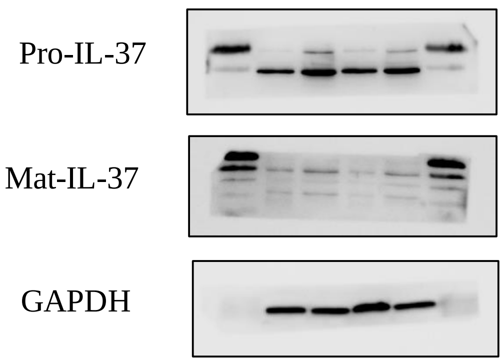

Name of lanes: Control, IL-1β, Control , IL-1β

P1 P7

Two other images for quantitative analysis

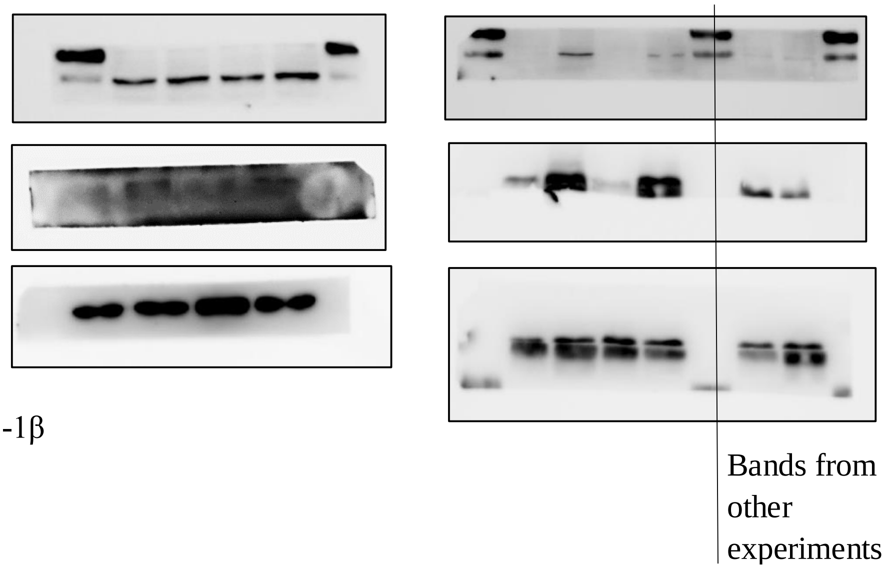

Figure. 2C

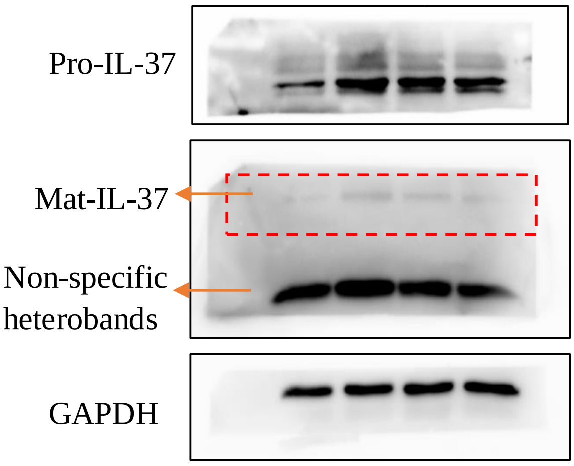

Name of lanes: Control, IL-1β, HMGB1, LPS

Two other images for quantitative analysis

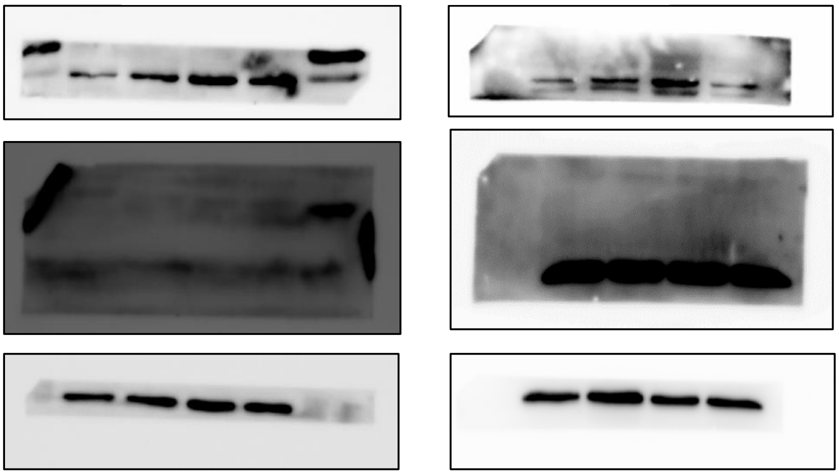

Figure. 3A

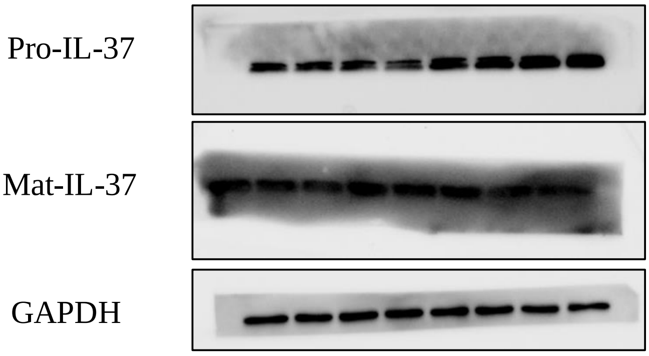

Name of lanes: 0, 3, 6, 9, 12, 24, 36, 48h,

Figure. 3B

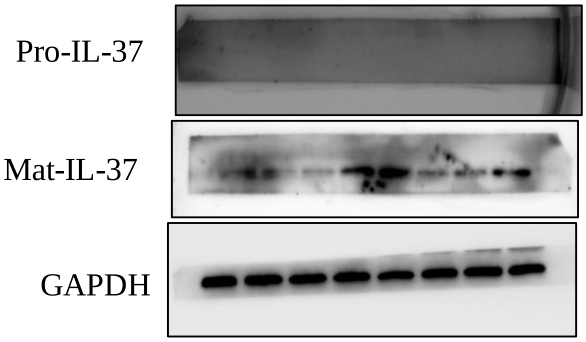

Name of lanes: 0, 3, 6, 9, 12, 24, 36, 48h,

Two other images for quantitative analysis

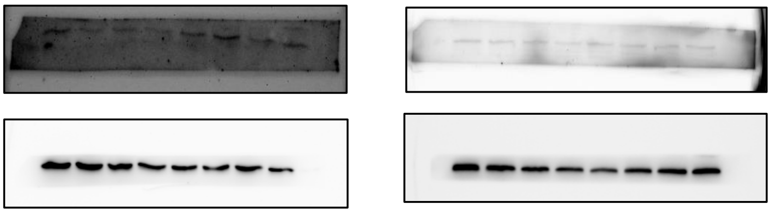

Figure. 4B

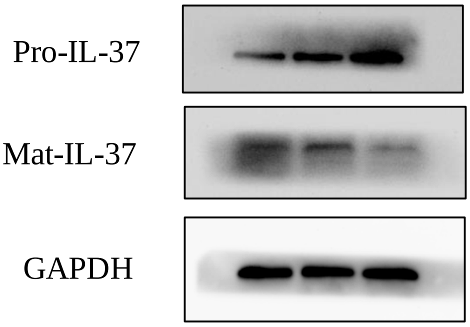

Name of lanes: Control, IL-1β, IL-1β+VX-765

Two other images for quantitative analysis

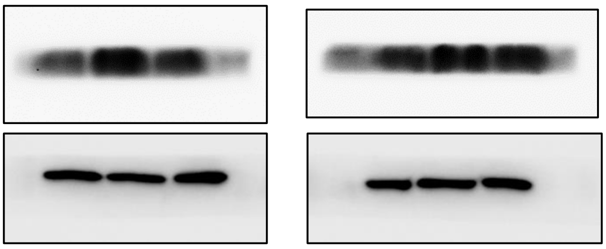

Figure. 4D

Two other images for quantitative analysis

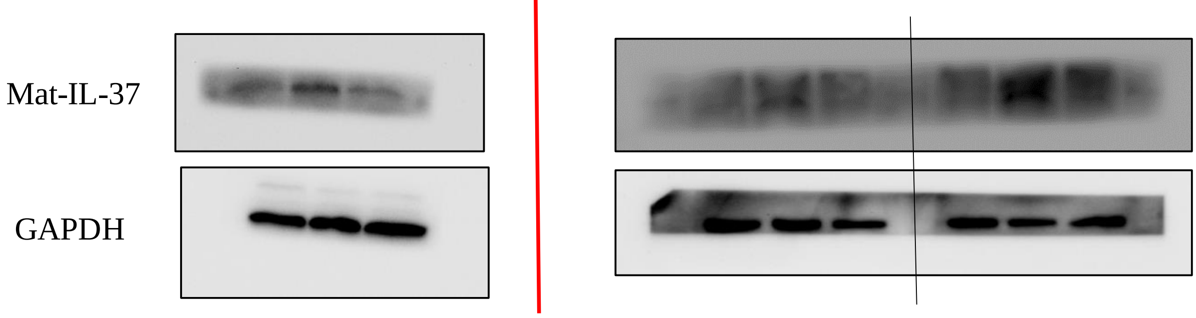

Name of lanes: Control, IL-1β, IL-1β+VX-765

Figure. 4G

Two other images for quantitative analysis

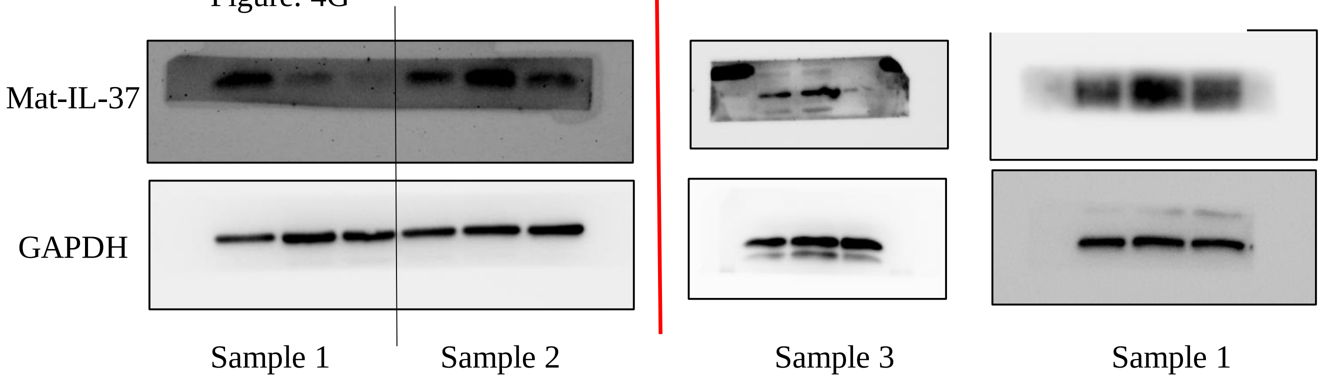

Name of lanes: Control, IL-1β, IL-1β+SIS3

Figure. 5B

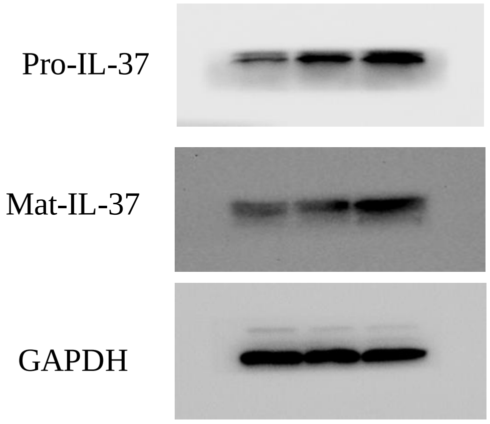

Name of lanes: Control, IL-1β+Lv-U6, IL-1β+Lv-37

Figure. 5D

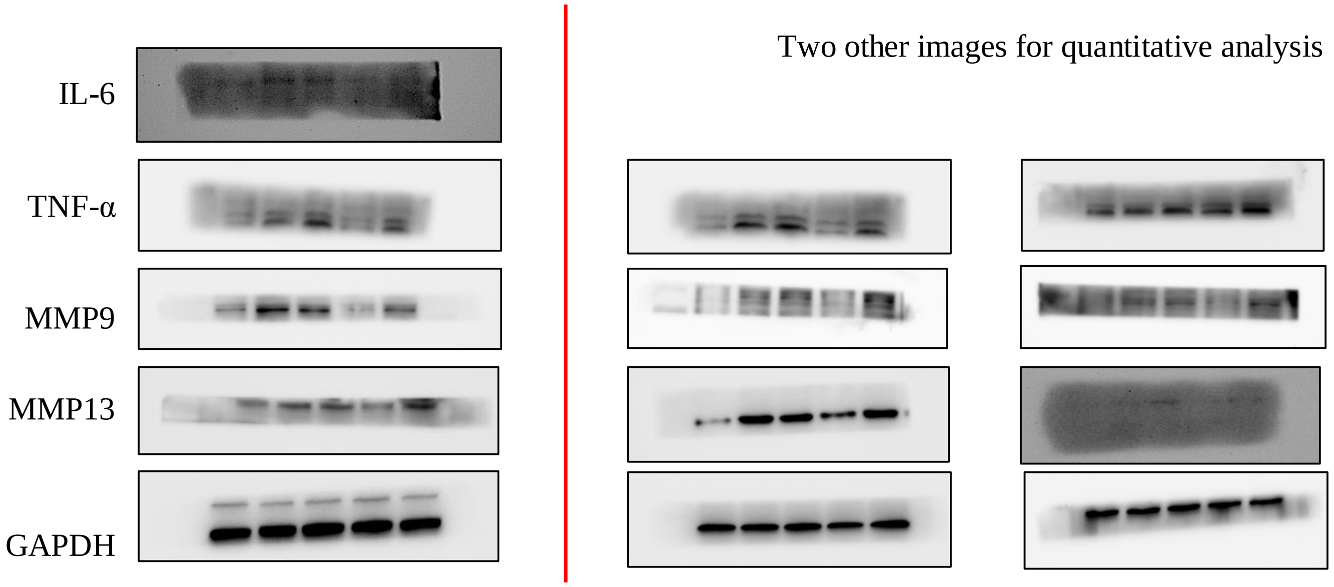

Name of lanes: Control, IL-1β, IL-1β+SIS3, IL-1β+Lv-IL-37, IL-1β+Lv-IL-37+SIS3
