# Supplementary figures and images for: IL-37 counteracts inflammatory injury in the temporomandibular joint via the intracellular pathway
Source: Front Pharmacol. 2023 Nov 20;14:1250216. doi: 10.3389/fphar.2023.1250216 (PMC10694265; doi:10.3389/fphar.2023.1250216)

Fig. 1C

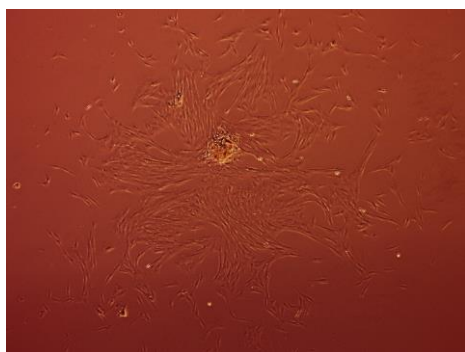

Fig. 1D

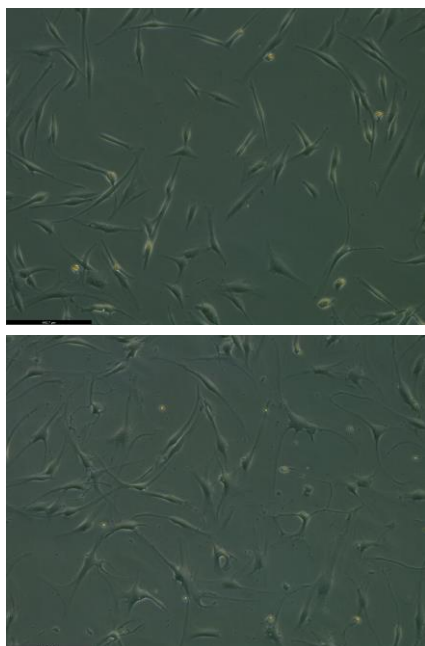

Fig. 1E

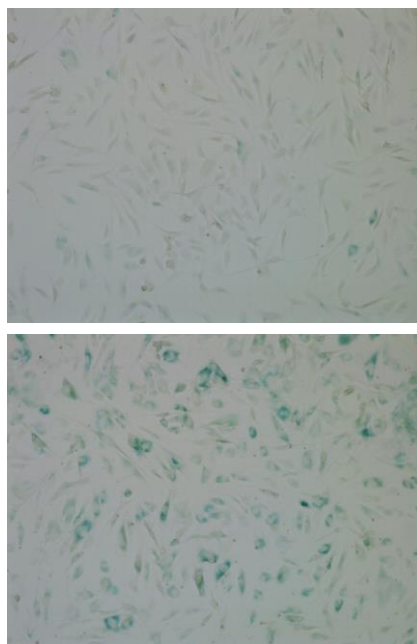

Fig. 4F

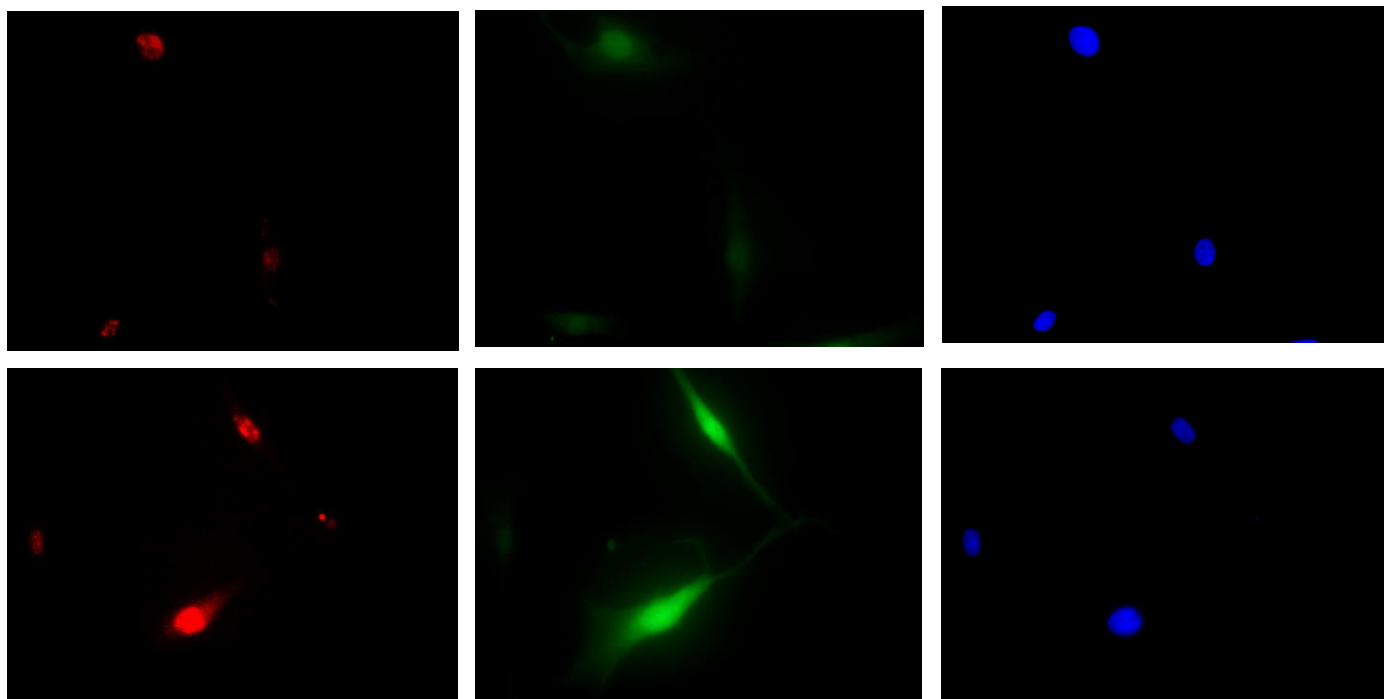

Supplement: Supplementary file 1 [file DataSheet1.ZIP › original image.pdf]
